# Association between *MMP-3* polymorphisms among Chinese patients with osteonecrosis of the femoral head

**DOI:** 10.18632/oncotarget.22313

**Published:** 2017-11-06

**Authors:** Yuxin Qi, Yong Zhu, Yuju Cao, Huiqiang Wu, Mingqi Sun, Hao Wu, Linlin Pan, Guoqiang Wang, Jianzhong Wang

**Affiliations:** ^1^ Inner Mongolia Medical University, Hohhot, Inner Mongolia 010050, China; ^2^ Department of Orthopedics and Traumatology, The 2nd Affiliated Hospital of Inner Mongolia University, Hohhot, Inner Mongolia 010030, China; ^3^ Zhengzhou TCM Traumatology Hospital, Zhengzhou, Henan 450016, China; ^4^ Inner Mongolia Autonomous Region Hospital of Traditional Chinese Medicine, Hohhot, Inner Mongolia 010000, China

**Keywords:** case-control study, MMP3, single-nucleotide polymorphism, osteonecrosis of the femoral head

## Abstract

Many potential causative factors are related to the initiation and progression of osteonecrosis of the femoral head (ONFH). The matrix metalloproteinase/tissue inhibitor of metalloproteinases (MMPs/TIMPs) system was found to play a significant role in the development of ONFH. The aim of this study is to investigate the association between polymorphisms of *MMP-3* and ONFH in the Chinese population. We selected 8 single-nucleotide polymorphisms (SNPs) in 2 genes selected from the *MMPs/TIMPs* system in a case–control study with 585 cases of ONFH and 507 healthy controls. Odds ratios (ORs) and 95% confidence intervals (CIs) were estimated using the chi-squared test, genetic model analysis, haplotype analysis, and stratification analysis. We found that the minor allele of rs650108 and rs522616 (*p*<0.05) was assumed a risk allele compared to the wild-type allele. In the genetic model analysis, We observed two susceptibility SNPs additionally: rs650108, dominant model analyses (with adjustment: OR=0.73; 95%CI 0.56-0.95; p=0.017) and additive model analyses (with adjustment: OR=0.83; 95%CI 0.70-0.99; p=0.044); and rs522616 recessive model analyses (with adjustment: OR=1.52; 95%CI 1.07-2.14; p=0.018) and additive model analyses (with adjustment: OR=1.21; 95% CI 1.02-1.44; p=0.033). Our results verify that genetic variants of *MMP3* contribute to ONFH susceptibility in the population of northern China. In addition, we found that gender differences might interact with *MMP3* polymorphisms to contribute to the overall susceptibility to ONFH.

## INTRODUCTION

Osteonecrosis of the femoral head (ONFH) is a debilitating bone disease in which patients experience the collapse of the joint cartilage and femoral head and a subsequent loss of joint function caused by abnormities in the fibrinolytic system and a disorder in the blood supply [1–3]. Since this stubborn disease may seriously affect the quality of life for patients, a large number of researchers have kept an eye on the identification of risk factors for ONFH. A great deal of ONFH cases develop in association with alcohol intake and steroid therapy are also contributing factors. Though it is well known that ONFH is caused by many factors, genetic factors have been demonstrated to be a strong factor of this disturbance. Many candidate genes have been shown related to ONFH in previous studies [4].

Repair ability and bony remodeling play a significant part in the development and severity of ONFH, nevertheless, little is known about the potential regulatory and repair machine-processed [5, 6]. Matrix metalloproteinases (MMPs), an enzyme family that provides the extracellular matrix (ECW) remodeling, play an important parts in the tissue remodeling process and physiological and pathological repair [7, 8]. MMPs and tissue inhibitor of matrix metalloproteases (TIMPs) expression and activity that may increase the ability to repair damaged bone matrix influencing the equilibrium between bone resorption and de novo bone formation in ONFH.

The ECW host some structural molecules (protein, proteoglycans, polysaccharides) as well as some enzymes, both being secreted by certain cells forming a 3-dimensional macromolecule network specific to different tissues, in a way to create cellular microenvironments or niches. In case the regulation of ECW remodeling is lost, tissue integrity is jeopardized, leading to development of pathological processes including connective tissue disorders, cancer, and metastasis (tumor microenvironment) [9, 10]. Matrix metalloproteinases (MMPs), an enzyme family that provides the ECW remodeling, are responsible for degradation of ECW elements. In biology, MMPs have been linked to ECM degradation and turnover [11, 12]. Though certain MMPs are expressed in bone and cartilage tissue during the normal bone development, MMPs-2,-9,-13,-14, and-16 play an essential part in skeletal development, as shown by knockout mice models and human genetic diseases [13, 14]. MMP-3 is a part of the stromelysin which is expressed in few cell types that contain human articular chondrocytes and synoviocytes [15]. According to reports in the literature, Other MMPs can proteolytically activated by MMP-3, and pathological conditions might ensue by means of the *MMP* genes overexpression [16].

Studies have demonstrated association of *MMP-3* polymorphism with knee osteoarthritis and osteosarcoma [17, 18]. Nevertheless, previous studies have rarely investigated the association between genetic variants in MMP-3 and the risk of ONFH. We conducted a case-control study to analyze the relevance between 8 single nucleotide polymorphisms (SNPs) in *MMP-3* and the risk of ONFH in a Chinese Han population.

## RESULTS

A total of 585 cases and 507 controls were included in this study. The demographic in ONFH cases and control are shown in Table 1. As shown in Table 2, the Sequenom MassARRAY Assay Design 3.0 Software was used to design a multiplexed SNP MassEXTEND assay. All 8 single nucleotide polymorphisms were checked for Hardy Weinberg equilibrium in the control group (Table 3). We used chi square test to compare the allelic frequency distributions between cases and controls.

**Table 1:** Characteristics of cases and controls in this study

Variable(s)	Case (n=585)	Control (n=507)	*p* value
Sex N(%)			>0.05^a^
Male	472(80.7)	396(78.1)	
Female	113(19.3)	111(21.9)	
Age, years (mean ± SD)	42.61±12.95	47.43±9.74	<0.001^b^

*p* ≤ 0.05 indicates statistical significance.

^a^ Two-sided Chi-squared test.

^b^ Independent samples *t* test.

**Table 2:** Primers Used for this Study

SNP_ID	1st-PCRP	2nd-PCRP	UEP_SEQ
rs639752	ACGTTGGATGCA GATAAATTCTCCACTTGC	ACGTTGGATGGGCT GCAATGCAGGGAAAAG	tGGGAAGAAAGA AATAGGTGAT
rs650108	ACGTTGGATGGTC ACTGTCTCATTGTGTGT	ACGTTGGATGTCAGG TAGAGGTGACAAGTG	tAAGTGGGT GAGGTTAGA
rs520540	ACGTTGGATGGCG AAAGGGCTTAACTGTTAT	ACGTTGGATGCCA GCTCGTACCTCATTTCC	CTCGTACCT CATTTCCTCTGAT
rs646910	ACGTTGGATGCCA CTGTAAGCTGGTGACTA	ACGTTGGATGGTTA AGCCCTTTCGCTTTAG	CGCTTTAGAAA TACACTTTAGCATCT
rs602128	ACGTTGGATGCT TCGGGATGCCAGGAAA	ACGTTGGATGAAG CTGGACTCCGACACTCT	CAGGTGTG GAGTTCCTGA
rs679620	ACGTTGGATGAACA GGACCACTGTCCTTTC	ACGTTGGATGAGA AATATCTAGAAAACTAC	tcTCTAGAAAAC TACTACGACCTC
rs678815	ACGTTGGATGAATG CAACGTAATTTTAGC	ACGTTGGATGTGGA GTATTTCTCTAGCTTG	TCTCTAGCTTG CTGAAATAATG
rs522616	ACGTTGGATGCGTA GCTGCTCCATAAATAG	ACGTTGGATGACAGAG AGAATTTCAGTCCG	gaCGGTAAGCAA TGTAATTCATTTCA

**Table 3:** Allele frequencies in cases and controls and odds ratio estimates for ONFH

SNP ID	Gene	Position	Alleles A/B	MAF		*p*^a^ value for HWE	ORs	95% CI		*p*^b^
				Case	Control					
rs639752	MMP3	11q22.2	C/A	0.32	0.35	0.116	0.88	0.74	1.06	0.177
rs650108	MMP3	11q22.2	G/A	0.39	0.44	0.147	0.84	0.70	0.99	0.040^*^
rs520540	MMP3	11q22.2	A/G	0.32	0.35	0.116	0.88	0.74	1.06	0.177
rs646910	MMP3	11q22.2	A/T	0.07	0.09	1	0.82	0.60	1.13	0.224
rs602128	MMP3	11q22.2	A/G	0.32	0.34	0.235	0.90	0.75	1.07	0.234
rs679620	MMP3	11q22.2	T/C	0.32	0.35	0.202	0.88	0.74	1.05	0.156
rs678815	MMP3	11q22.2	G/C	0.32	0.35	0.141	0.88	0.74	1.05	0.165
rs522616	MMP3	11q22.2	C/T	0.40	0.35	0.923	1.20	1.00	1.42	0.044^*^

SNP single nucleotide polymorphism, HWE Hardy-Weinberg equilibrium, OR odds ratio, 95% CI 95% confidence interval, MAF minor allele frequency.

^*^ *p* ≤ 0.05 indicates statistical significance.

^a^ *p* was calculated by exact test.

^b^*p* was calculated by Pearson Chi-squared test.

As a result, we discovered that rs650108 and rs522616 were associated with ONFH risk in the *MMP3* (rs650108 *p* = 0.040, OR=0.83; 95 % CI:0.70–0.99 and rs522616 *p* = 0.044, OR=1.20; 95 % CI:1.01–1.42). A rigorous Bonferroni correction analysis was applied so as to reduce the potential of spurious findings due to multiple testing. Nevertheless, the difference was no longer significant after Bonferroni correction. In contrast to wild-type alleles, the minor allele of each SNP was assumed a risk allele. We have listed the Minor allele frequency (MAF) in cases and controls in Table 3.

**Figure 1:**
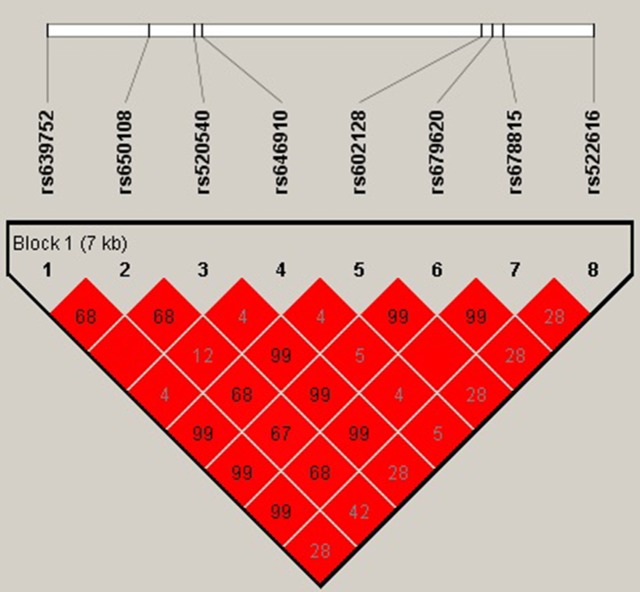
Linkage disequilibrium (LD) plots containing 8 SNPs from MMP3 Red squares display statistically significant associations between a pair of SNPs, as measured by r^2^; darker shades of red indicate higher r^2^.

As listed in Table 4, we compared the risk of ONFH and the SNP genotypes. We identified the association between two significant SNP genotypes and the risk of ONFH. We observed two susceptibility SNPs additionally under two models respectively: rs650108, dominant model (with adjustment: OR=0.73; 95% CI:0.56-0.95; *p* = 0.017) and additive model(with adjustment: OR=0.83; 95% CI:0.70-0.99; *p* = 0.044); and rs522616 recessive model(with adjustment: OR=1.52; 95% CI:1.07-2.14; *p* = 0.018) and additive model (with adjustment: OR=1.21; 95% CI:1.02-1.44; p = 0.033). We use linkage disequilibrium (LD) and haplotype analyses to characterize the SNPs in MMP3. We calculated LD between 8 SNPs and the haplotype structure of the MMP3 gene was analyzed (r^2^). However, the significant difference was not found in haplotypes analysis. LD blocks were detected in the control group (Figure 1).

**Table 4:** Genotypic model analysis of relationship between SNPs and ONFH risk

SNPs	Model	Genotype	Group=control	Group=hormone	Without adjustment		With adjustment		AIC	BIC
					OR (95% CI)	*p*^*a*^-value	OR (95% CI)	*p*^*a*^-value		
rs650108	Codominant	A/A	153 (30.3%)	216 (37%)	1.00	0.065	1.00	0.058	1461.8	1486.8
		A/G	265 (52.5%)	279 (47.8%)	0.75 (0.57-0.97)		0.73(0.55-0.96)			
		G/G	87 (17.2%)	89 (15.2%)	0.72 (0.51-1.04)		0.74 (0.51-1.07)			
	Dominant	A/A	153 (30.3%)	216 (37%)	1.00	0.02^*^	1.00	0.017^*^	1459.8	1479.8
		A/G-G/G	352 (69.7%)	368 (63%)	0.74 (0.57-0.95)		0.73(0.56-0.95)			
	Recessive	A/A-A/G	418 (82.8%)	495 (84.8%)	1.00	0.37	1.00	0.5	1465	1485
		G/G	87 (17.2%)	89 (15.2%)	0.86 (0.63-1.19)		0.89 (0.64-1.24)			
	Overdominant	A/A-G/G	240 (47.5%)	305 (52.2%)	1.00	0.12	1.00	0.08	1462.4	1482.4
		A/G	265 (52.5%)	279 (47.8%)	0.83 (0.65-1.05)		0.80 (0.63-1.03)			
	Log-additive	—	—	—	0.83 (0.70-0.99)	0.037^*^	0.83 (0.70-1.00)	0.044^*^	1461.5	1481.4
rs522616	Codominant	T/T	212 (41.9%)	224 (38.3%)	1		1	0.052	1464.3	1489.2
		T/C	230 (45.5%)	259 (44.3%)	1.07 (0.82-1.38)	0.078	1.08 (0.83-1.40)			
		C/C	64 (12.7%)	102 (17.4%)	1.51 (1.05-2.17)		1.57(1.08-2.29)			
	Dominant	T/T	212 (41.9%)	224 (38.3%)	1	0.23	1	0.19	1466.4	1486.4
		T/C-C/C	294 (58.1%)	361 (61.7%)	1.16 (0.91-1.48)		1.18 (0.92-1.52)			
	Recessive	T/T-T/C	442 (87.3%)	483 (82.6%)	1	0.027^*^	1	0.018^*^	1462.6	1482.5
		C/C	64 (12.7%)	102 (17.4%)	1.46 (1.04-2.05)		1.52(1.07-2.14)			
	Overdominant	T/T-C/C	276 (54.5%)	326 (55.7%)	1	0.7	1	0.69	1468	1488
		T/C	230 (45.5%)	259 (44.3%)	0.95 (0.75-1.21)		0.95 (0.75-1.21)			
	Log-additive	—	—	—	1.19 (1.00-1.41)	0.048^*^	1.21(1.02-1.44)	0.033^*^	1463.6	1483.6

^*^p ≤ 0.05 indicates statistical significance.

*p* values were calculated by Wald test by unconditional logistic regression adjusted for age and gender.

AIC, Akaike’s Information criterion; BIC, Bayesian Information criterion.

## DISCUSSION

We researched the associations between 8 SNPs in the *MMP3* gene and the risk of ONFH. In this case-control study, we confirmed for the first time that MMP3 genetic polymorphisms (rs650108 and rs522616) were associated with a risk of ONFH. We can also observed that a protective effect for the dominant model “A/G-G/G” of rs650108 the *MMP3* gene was related to a reduction in the risk of developing ONFH. Furthermore, it can be found that a strong effect of the “C/C” recessive model of rs522616 in the *MMP3* can increase the risk of developing ONFH.

It is well know that the SNP occurring in *MMP* gene promoters can affect the expression of MMPs [19]. MMP3, a member of the family of endogenous proteolytic enzymes, is produced by chondrocytes and can degrade many extracellular matrix components except glucose [20]. Previous study has shown that the dynamic equilibrium between the Timp3 and Mmp3 is broken, the inhibition of MMP3 by TIMP3 would be reduced, and the degradation of matrix proteins would be increased in the model rats. It also shows that the development of osteoarthritis can be influenced by the destruction of the balance between MMP3 and TIMP3 [20]. In addition, cartilage degeneration may happen during latestage steroid-induced osteonecrosis of the femoral head, after bone tissue collapse [21].Thus, we postulate that MMP3 is related to ONFH.

In our study, we investigated eight SNPs in *MMP3* (in Table 3). Among these SNPs, the rs522616 and rs650108 polymorphisms of *MMP-3* have been identified in different diseases, such as chronic periodontitis and sporadic brain arteriovenous malformation [22, 23]. It is not completely determined whether the rs522616 and rs650108 polymorphisms of *MMP-3* can influence the susceptibility or severity in patients with ONFH. Therefore, it has been hypothesized that the genetic variations in *MMP3* can influence the susceptibility to ONFH. In our study, we only found that the SNPs of the rs522616 and rs650108 are associated with a risk of ONFH. As far as we know, we are the first to report the relation between *MMP-3* polymorphisms rs522616, rs650108 and ONFH risk, but the conclusion identified should be proved in further studies.

There are important discoveries revealed in our study, but some limitations of this study should be considered when interpreting these results. First of all, our study does not include an analysis of biological functions, which will be crucial for elucidating the role of MMP3 in ONFH. Secondly, risk factors for ONFH can be classified into different clinical causes for further analysis. Thirdly, the participants in our study were all Han Chinese individuals recruited from the Zhengzhou Traditional Chinese Medicine Traumatology Hospital, which might involve a selection bias. Fourthly, we used a hospital-based case–control design, which may involve selection bias. Finally, the sample size was relatively small after stratification by sex, which might convert the positive findings into negative results. A larger case–control study is expected to circumvent those problems, which could make our conclusions more powerful.

To sum up, we have confirmed for the first time that 2 susceptive SNPs (rs522616 and rs650108) of *MMP3* from the MMPs/TIMPs system exhibit a significant association with increased risk of ONFH in the population of northern China. Further functional studies and larger population-based studies are needed to confirm our results.

## MATERIALS AND METHODS

### Ethics statement

The use of human tissue and the protocol in this study were abided by the principles of the Declaration of Helsinki and were approved by the Ethical Committee of Zhengzhou Traditional Chinese Medicine Traumatology Hospital. All candidate subjects signed informed consent.

### Study population

We recruited a total of 585 patients diagnosed with ONFH and 507 control subjects were consecutively enrolled from 2014 to 2015 among Han Chinese. All the subjects were treated by the Affiliated Zhengzhou Traditional Chinese Medicine Traumatology Hospital. All cases were verified, and patients were recruited without age, sex, or disease stage restriction. Moreover, patients did not receive systemic inflammatory treatment including drug control treatment before the blood samples used in this study were obtained.

A number of 507 healthy unrelated individuals were recruited randomly as sample, and the participants were Han Chinese living in Zhengzhou city and nearby. All of the chosen subjects were from the Zhengzhou Traditional Chinese Medicine Traumatology Hospital. To reduce the potential environmental and therapeutic factors impacting the variation of complex human diseases, we performed detailed recruitment and set exclusion criteria to exclude subjects with diseases related to genetic susceptibility, such as tumor.

### SNP selection and genotyping

We selected 8 SNPs for investigation in this study. We prioritized SNPs to be studied considering: (a) previous reports of expression in diseased tissues, (b) previous reports of association with ONFH, (c) substrates as recognized molecules in diseased tissues.

Within selected SNPs, 8 polymorphisms were selected based on published reports and/or their locations in the genes, based on their likelihood to have functional consequences (i.e., located in the promoters, exons or near exon/intron boundaries), or if considered tag-SNPs as surrogates for the linkage disequilibrium blocks surrounding the candidate gene. We used information from the NCBI dbSNP (http://www.ncbi.nlm.nih.gov/snp) and the HapMap Project (http://www.hapmap.org) databases.

A total of 8 tSNPs in the MMP3 gene were selected for further genotyping. The phenol–chloroform extraction method was performed to extract genomic DNA from whole blood [24]. DNA concentration was measured by spectrometry (DU530 UV/VIS spectrophotometer, Beckman Instruments, Fullerton, CA, USA). Sequenom MassARRAY Assay Design 3.0 software was used to design multiplexed SNP MassEXTEND assay, and SNP genotyping was performed utilizing the Sequenom MassARRAY RS1000 recommended by the manufacturer [25]. Data management and analyses were performed using the Sequenom Typer 4.0 software as previouslydescribed [25, 26].

### Statistical analysis

We used Microsoft Excel and SPSS 16.0 (SPSS, Chicago, IL, USA) to perform statistical analyses. In this study, all p values were two-sided, and p ≤ 0.05 was considered as achieving the threshold of statistical significance. Observed genotype frequencies were compared with expected frequencies to test for deviations from Hardy–Weinberg equilibrium (HWE). Chi-squared test/Fisher’s exact test was used to calculate the allele and genotype frequencies of cases and controls [27]. ORs and 95% CIs were calculated by unconditional logistic regression analyses adjusted for age and sex [28]. The possibility of sex differences as a source of population substructure was evaluated by a genotype test for each SNP in male and female, and the number of significant results at the 5 % level was compared with the number expected by the Chi-squared test [27]. The five genetic models (codominant dominant, recessive overdominant and log-additive) were applied by PLINK software (http://pngu.mgh.harvard.edu/purcell/plink/) to assess the association of single tSNPs with the risk of ONFH. ORs and 95% CIs were calculated by unconditional logistic regression analyses adjusted for age and sex [28, 29] At last, we use the Haploview software package (version 4.2) [30]to evaluate LD patterns and haplotypes.

## Abbreviations

CI: confidence interval
HWE: Hardy–Weinberg equilibrium
MMPs: matrix metalloproteinases
OA: osteoarthritis
ONFH: osteonecrosis of the femoral head
OR: odds ratio
SNPs: single-nucleotide polymorphisms
